# Endovascular Embolization of Intracranial Aneurysms Using Target Tetra Detachable Coils: Angiographic and Clinical Results from a Single Center

**DOI:** 10.3390/jcm13164940

**Published:** 2024-08-21

**Authors:** Wook Kim, Tae Keun Jee, Je Young Yeon, Keon Ha Kim, Jong-Soo Kim, Pyoung Jeon

**Affiliations:** 1 Department of Radiology, Samsung Medical Center, Sungkyunkwan University School of Medicine, Seoul 06351, Republic of Korea; wugawuga88@gmail.com (W.K.); somatom.kim@samsung.com (K.H.K.); 2 Department of Neurosurgery, Samsung Medical Center, Sungkyunkwan University School of Medicine, Seoul 06351, Republic of Korea; taekeun.jee@samsung.com (T.K.J.); yeonjay.youn@samsung.com (J.Y.Y.); jsns.kim@samsung.com (J.-S.K.)

**Keywords:** endovascular coil embolization, intracranial aneurysm, target tetra detachable coil

## Abstract

**Background/Objectives**: Target tetra detachable coils (TTDCs) aid in achieving effective framing during the coil embolization of small intracranial aneurysms by maintaining a tetrahedral conformation within the aneurysm sac. We aimed to report the initial experience of patients treated for intracranial aneurysms using TTDCs, with a specific focus on efficacy and safety. **Methods**: We retrospectively reviewed the medical records of 41 patients who underwent the coil embolization of intracranial aneurysms sized ≤10 mm with TTDCs between April and May 2023. Post-procedural angiographic and clinical results were reviewed. **Results**: Of the 46 aneurysms (45 unruptured and 1 ruptured), 33 (71.7%) were treated with the stent-assisted technique and 13 (28.3%) using the simple coil embolization technique. Post-procedural angiography showed complete occlusion in 41 aneurysms (89.1%), neck remnants in 1 (2.2%), and residual aneurysms in 4 (8.7%). The mean packing density was 34.7% (19.3–46.8%), with TTDC coil length comprising a mean of 88.5% of the total coil length. No major device- or procedure-related complications were observed. During the follow-up, 40 aneurysms (93.0%) demonstrated complete occlusion, while neck remnants were observed in 1 (2.3%), and residual aneurysms in 2 (4.7%). No cases of recanalization were observed. **Conclusions**: The TTDC is a safe and effective device for the endovascular treatment of intracranial aneurysms. Follow-up studies are required to establish long-term results.

## 1. Introduction

Since the introduction of Guglielmi detachable coils (GDCs) in clinical neuroendovascular practice, endovascular coil embolization has remained the primary treatment modality for ruptured and unruptured intracranial aneurysms [1,2]. Advancements in coil-assistance devices over the past decade, including balloon catheters and stents, have expanded the range of aneurysms that can now be treated [3,4,5,6,7,8,9]. Especially, the introduction of low-profile stents such as the LVIS Jr. (MicroVention-Terumo, Inc., Tustin, CA, USA) and the Neuroform Atlas (Stryker Neurovascular, Fremont, CA, USA), which are compatible with 0.017-inch microcatheters, has enabled the treatment of more distal aneurysms that were previously difficult to manage [10,11,12]. Moreover, several newer flow-diverting stents, including the Surpass Evolve (Stryker Neurovascular, Fremont, CA, USA) and the Pipeline Vantage (Medtronic, Irvine, CA, USA), which are compatible with 0.027-inch microcatheters and designed to enhance safety and efficacy profiles, are now commercially available [13,14]. The flow disruptor, Woven EndoBridge (WEB; Sequent Medical, Aliso Viejo, CA, USA), is a barrel-shaped, self-expanding device constructed from nitinol wires, developed specifically for treating wide-neck bifurcation aneurysms. Several studies have demonstrated promising safety and efficacy outcomes with the WEB for both ruptured and unruptured intracranial aneurysms [13,14,15,16]. Despite these advances in the endovascular treatment of intracranial aneurysms, coil embolization, including simple coil embolization and stent-assisted coil embolization, remains fundamental in treating intracranial aneurysms.

Dense coil packing of the intracranial aneurysm is necessary for successful embolization and the prevention of recurrence. Therefore, the proper selection of coils, taking into account the aneurysm size and shape, should be considered during the specific coil embolization phase [17]. Small intracranial aneurysms are technically challenging and harbor increased risks of rupture compared with larger aneurysms [18,19,20,21]. Such difficulties stem from the challenges in selecting aneurysms using microcatheters and choosing appropriate coils to maintain conformation in a limited space, especially during the framing process. Coil herniation into the parent artery poses an additional risk of thromboembolic events, often necessitating the deployment of a stent to stabilize the coil mass and ensure proper packing density within the aneurysm. Despite these challenges, endovascular treatment for small intracranial aneurysms has advanced considerably and is considered a successful treatment choice.

**Figure 1:**
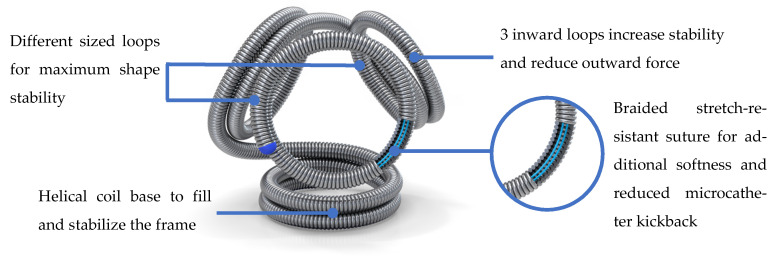
Target tetra detachable coil (TTDC). By maintaining a tetrahedral conformation inside an intracranial aneurysm, it is feasible to treat small aneurysms, which is advantageous, especially during the framing process. In addition, a braided stretch-resistant suture allows for a smoother delivery and reduced microcatheter kickback.

Recently, a target tetra detachable coil (TTDC) (Stryker Neurovascular, Fremont, CA, USA) was introduced (Figure 1). By maintaining a tetrahedral conformation inside an intracranial aneurysm, it is feasible to treat small aneurysms, which is advantageous, especially during the framing process. In addition, a braided stretch-resistant suture allows for a smoother delivery and reduced microcatheter kickback. Therefore, the coil is ideal not only for the framing process but also advantageous during the filling and finishing steps. TTDCs are expected to maintain a stable conformation during the procedure, potentially reducing the need for stent usage in small wide-neck aneurysms. This is particularly advantageous in terms of decreasing the risk of thromboembolic complications. 

The use of TTDCs for treating cerebral aneurysms has been conducted at a limited number of centers in South Korea, and to the best of our knowledge there are currently no studies evaluating the efficacy and safety of TTDCs. We aimed to report the initial experience of patients treated for intracranial aneurysms using TTDCs, with a specific focus on safety and efficacy.

## 2. Materials and Methods

This research was granted approval by the Institutional Review Board (IRB File No. 2023-07-115) of Samsung Medical Center, Seoul, Republic of Korea. All patients or their legal representatives provided written informed consent for both treatment and data collection.

### 2.1. Patient Selection and Data Collection

We retrospectively reviewed the medical records of patients who underwent intracranial aneurysm coil embolization at our center between April and May 2023. In cases in which the specific sizes of the TTDCs were not available, coils from other vendors were used. Inclusion criteria were as follows: (1) Confirmed diagnosis of one or more intracranial aneurysms; (2) Aneurysm (either ruptured or unruptured) with a size <10 mm in maximal diameter; (3) Procedures in which TTDCs comprised more than 50% of the total coil length. The exclusion criterion was the presence of aneurysms associated with arteriovenous malformations or dissection. 

Clinical information, including age, sex, medical history, and platelet reactivity test outcomes was gathered from the patients’ medical records. The size of the aneurysm, neck diameter, and dome-to-neck (DNR) ratio were assessed by three-dimensional digital subtraction angiography and confirmed based on the maximum size in each dimension. The aneurysm location and incorporated branch were also recorded, and the final assessment of the parent artery and branch vessel patency was evaluated using post-procedural angiography. Packing density was assessed by applying the following equation, which measures the ratio between the volume of inserted coils and the volume of the aneurysm:

[coil length × π × (coil diameter/2)2]/[4π/3 × (d(1)/2) × (d(2)/2) × (d(3)/2)].

We also assessed the total number of coils used, TTDCs used, failed total coils, and failed TTDCs, in addition to the ratio of the length of the TTDC to the total coil.

### 2.2. Coil Embolization Procedure and Post-Procedural Evaluation

All procedures were performed under general anesthesia. Anesthesia was induced with a bolus intravenous injection of 2.0–2.5 mg/kg propofol and maintained with 6% sevoflurane inhalation at a concentration of 1.0 minimum alveolar concentration. Because of its safety and effectiveness, the combination of aspirin and clopidogrel has historically been the most commonly used dual antiplatelet regimen in the treatment of unruptured aneurysms [22]. All patients received premedication with a dual antiplatelet therapy regimen, which included either 100 mg of aspirin and 75 mg of clopidogrel for 5–14 days before the procedure. For ruptured aneurysms, dual antiplatelet premedication was not given, but a loading dose of dual antiplatelet medication (clopidogrel, 300 mg, and aspirin, 100–325 mg) was given to patients immediately after the procedure. The VerifyNow Assay (Accumetrics) was used to assess platelet reactivity in all patients. In clopidogrel hypo-responders (P2Y12 reaction units ≥ 230), ticlopidine (250 mg) twice a day was prescribed instead of clopidogrel. The activated coagulation time was maintained at 250–300 s during the procedure. Intravenous heparin infusion was continuously maintained for 24 h after the completion of the procedure for unruptured aneurysms, whereas heparin was stopped but not reversed for ruptured aneurysms. Dual antiplatelet medication was maintained for 3–6 months and then switched to aspirin monotherapy indefinitely. The duration of dual antiplatelet therapy was guided by findings from a recent nationwide cohort study, which indicated that 3 months of dual antiplatelet therapy was associated with a reduced risk of both ischemic and hemorrhagic complications [23]. 

Any type of coil embolization technique, including stent-assisted coil embolization, either by the jailing or stent-through technique, as well as simple embolization using either a single or double microcatheter technique, was determined based on the operator’s choice. The jailing technique involves inserting an aneurysm coiling microcatheter before stent deployment, whereas the stent-through technique refers to the insertion of the coiling microcatheter after stent deployment. In addition to packing density, the aneurysm’s angiographic occlusion rate was qualitatively assessed according to the Raymond and Roy Occlusion Classification (RROC) after the procedure as follows: Class I, complete occlusion; Class II, residual neck; and Class III, residual aneurysm [24]. 

Follow-up post-procedural imaging was performed during the hospitalization period using magnetic resonance imaging (MRI), which included both MR angiography and diffusion-weighted imaging (DWI). Based on radiological evaluation, diffusion-restricted lesions were classified as follows: (1) No lesion; (2) Focal dot-like lesion (size less than 1cm on DWI); (3) Territorial lesion (size more than 1cm on DWI). The radiological evaluation of aneurysmal occlusion based on RROC using MR angiography was obtained during the first follow-up visits at 6–12 months.

### 2.3. Outcomes

The primary outcome of this study was the initial rate of complete aneurysm occlusion according to RROC. RROC I and II closures are considered favorable angiographic findings, as there are reports supporting their good prognosis [25,26,27]. Secondary outcomes were angiographic occlusion rate according to RROC at first follow-up, rate of recanalization, rate of retreatment, any device- or procedure-related complications, including unexpected events such as puncture-related complications; device- or procedure-related major complications, defined as ischemic strokes or hemorrhages during the hospitalization period; and device- or procedure-related morbidity, defined as a recently developed neurological impairment after embolization. A major ipsilateral stroke was defined as an ipsilateral stroke characterized by an increase of ≥4 points on the National Institutes of Health Stroke Scale at 24 h after symptom onset, accompanied by territorial diffusion-restricted lesions detected by MRI. A Clinical Events Committee reviewed all serious events related to the device as well as pre-specified safety outcomes.

### 2.4. Statistical Analysis

Descriptive statistical analyses were carried out using clinical and radiographic factors, with the mean serving as a central tendency indicator. Statistical analyses were carried out using SPSS Statistics software (version 25.0; IBM Corp., Armonk, NY, USA).

## 3. Results

### 3.1. Patient Characteristics and Aneurysms

Table 1 and Table 2 summarize the patients’ characteristics and their aneurysms, respectively. A total of 41 patients with 46 intracranial aneurysms were analyzed. The mean patient age was 62.6 years, and 31 (75.6%) patients were female. The mean age of male patients was 57.2, while the mean age of female patients was 63.4. The mean maximum sac size was 4.5 mm (2.1–6.5 mm), and the mean neck size was 3.3 mm (1.6–6.1 mm). On average, the DNR was 1.3 (0.9–2.1), indicating wide-neck aneurysms. The most common aneurysm location was the internal carotid artery (*n* = 25, 54.3%), followed by the anterior cerebral artery (*n* = 9, 19.6%), posterior circulation (*n* = 7, 15.2%), and aneurysms involving the middle cerebral artery (*n* = 5, 10.9%). A total of 18 (39.1%) aneurysms were located at arterial bifurcations; 12 (26.1%) harbored incorporated branches; and 15 aneurysms (32.6%) contained lobule(s) on 3D rotational angiography. None of the 46 intracranial aneurysms were recurrent, and 1 (2.2%) was ruptured. The average aspirin reaction unit (ARU) and P2Y12 reaction unit (PRU) were 415 and 179, respectively. 

### 3.2. Procedural and Angiographic Data

**Figure 2:**
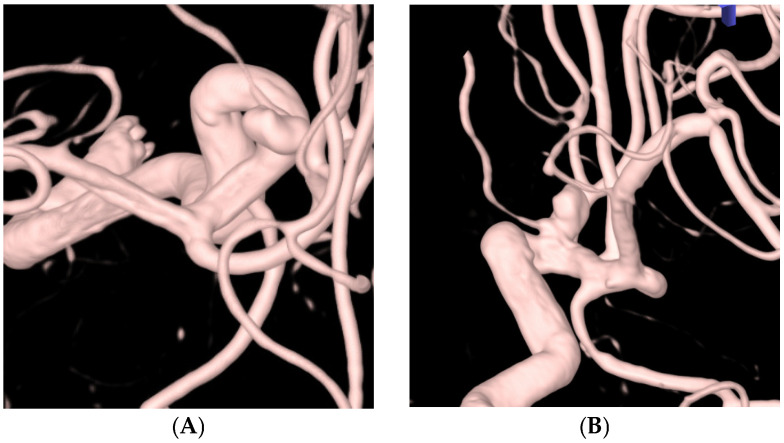

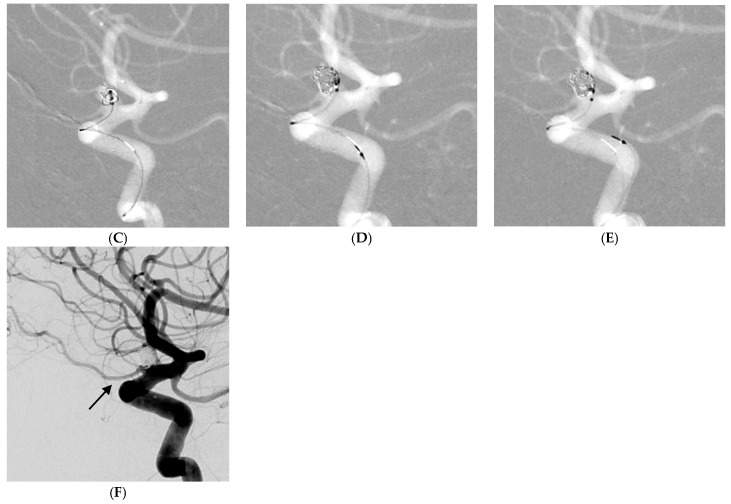
A 78-year-old female patient with a left ophthalmic artery aneurysm. (**A**,**B**) Three-dimensional rotational angiography shows a left ophthalmic artery aneurysm (neck: 3.7 mm, height: 4.4 mm, size: 6.2 × 4.2 mm). (**C**–**E**) The patient was treated with four coils including two target tetra detachable coils using a stent-assisted technique. (**F**) Postprocedural angiography showing complete occlusion of the aneurysm (Raymond and Roy Occlusion Classification I) with preservation of the left ophthalmic artery (arrow).

**Figure 3:**
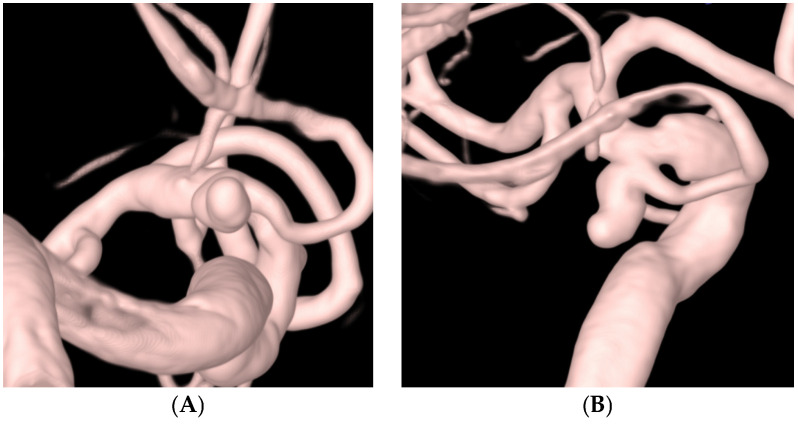

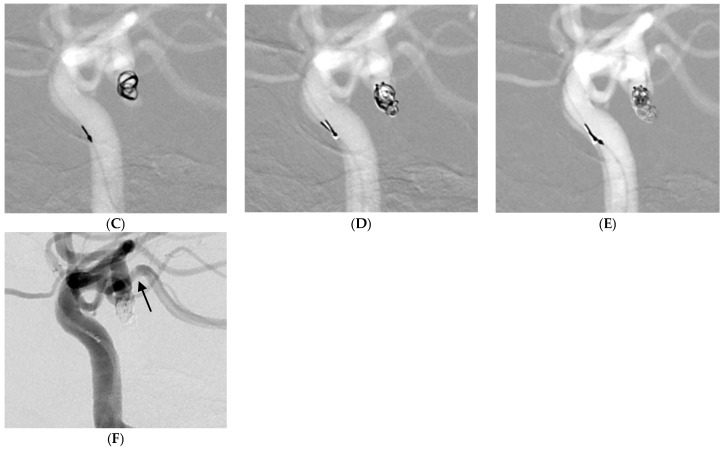
A 74-year-old female patient with a ruptured left posterior communicating artery aneurysm. (**A**,**B**) Three-dimensional rotational angiography shows a left posterior communicating artery aneurysm (neck: 3.1 mm, height: 4.7 mm, size: 3.9 × 3.5 mm). (**C**–**E**) The patient was treated with five target tetra detachable coils using a double microcatheter technique. (**F**) Postprocedural angiography showing complete occlusion of aneurysm (Raymond and Roy Occlusion Classification I) with preservation of the posterior communicating artery (arrow).

Table 3 outlines the procedural details and outcomes of this study. A total of 33 (71.7%) aneurysms were treated using the stent-assisted technique, and 13 (28.3%) were treated with the simple coil embolization technique. Of the 46 aneurysms, post-procedural angiography revealed complete occlusion in 41 (89.1%) (Figure 2 and Figure 3), neck remnants in 1 (2.2%), and residual aneurysms in 4 (8.7%). A favorable angiographic outcome was achieved in 42 aneurysms (91.3%). All 12 aneurysms harboring incorporated branches were completely preserved (Figure 2 and Figure 3). There was one case (2.2%) in which coil (TTDC) protrusion occurred during the procedure, which facilitated the use of an unplanned additional stent in the stent-assisted coil embolization group.

The average number of total coils used was 4.7 (range, 1–10), while the average number of TTDCs used was 3.9 (range, 1–10). In addition, the average number of total failed coils was 0.35 (range, 0–2), and the average number of failed TTDCs was 0.19 (range, 0–1). The mean coil packing density was 34.7% (range, 19.3–46.8%), and the average proportion of the total coil length used, represented by TTDC, was 88.5% (range, 52.2–100%). The packing density in the stent-assisted coil embolization group was 35.3% (range, 19.3–41.5%), while the packing density of those without stents was 33.2% (range, 24.3–46.8%).

No device- or procedure-related complications were observed. On post-procedural radiological evaluation, 34 (82.9%) patients showed no diffusion-restricted lesions, 7 presented with focal dot-like diffusion-restricted lesions, and territorial diffusion-restricted lesions were not observed. 

The first follow-up MR angiography was feasible for 43 aneurysms, with an average follow-up duration of 7.9 months (range, 5.5–12.5 months). Among these, 40 aneurysms (93.0%) showed complete occlusion, neck remnants were observed in 1 (2.3%), and 2 (4.7%) had residual aneurysms. A favorable angiographic outcome was achieved in 41 patients (95.3%). No instances of recanalization were observed, and no retreatments were required.

## 4. Discussion

We investigated the effectiveness of the TTDC for the treatment of small intracranial aneurysms. This retrospective series detailing the patients treated with TTDCs demonstrates adequate device efficacy and safety in both ruptured and unruptured aneurysms. Our results demonstrated favorable angiographic outcomes in 42 (91.3%) aneurysms treated in 46 patients using the TTDC alone or in combination with other coils. Notably, no device- or procedure-related complications were observed in all cases evaluated, and the achieved mean packing density was 34.7%. 

**Figure 4:**
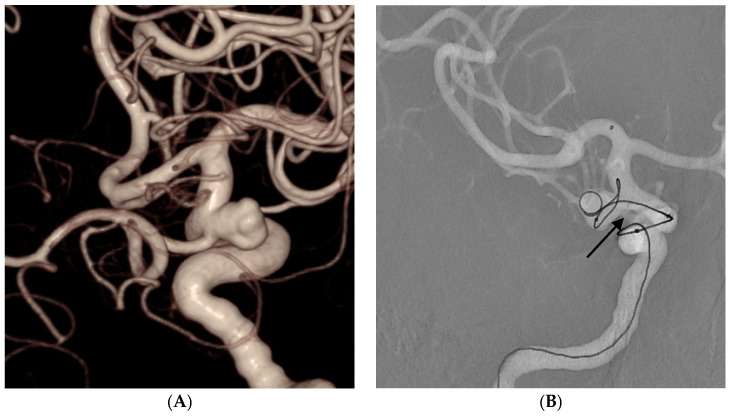
A 59-year-old male patient with an unruptured right posterior communicating artery aneurysm. (**A**) Three-dimensional rotational angiography shows a left posterior communicating artery aneurysm (neck: 5.0 mm, height: 5.8 mm, size: 7.8 × 5.1 mm). (**B**) The Target 360 Ultra coil of the appropriate size was initially selected as the framing coil. However, the coil expanded outside the aneurysmal sac and protruded into the parent artery, obliterating the posterior communicating artery (arrow). A new frame was established using the TTDC.

The TTDC exhibits promising characteristics for both frame formation and filling purposes. Its tetrahedral shape exhibited a tendency to occupy specific-sized spaces rather than spreading out into empty spaces, which was particularly evident during the deployment of the first and second coils. This facilitates successful frame formation and improves the preservation of the incorporated branches. Additionally, the TTDC consistently achieved a favorable packing density without compromising the integrity of the incorporated branches. Figure 2 and Figure 3 demonstrate the maintenance of the incorporated branches of the ophthalmic artery and the posterior communicating artery, respectively, following coil embolization using TTDCs. Compared to other target coils (Figure 4), where the frame coil tends to expand outside the aneurysmal sac and protrude into the parent artery, TTDCs tend to reside in the aneurysmal sac with a stable frame. However, generalization should bring caution since the morphological features of aneurysms differ between patients, and the selection of the appropriate coil size is as important as choosing different types of coils.

In the present study, the aneurysms were not only small but also exhibited wide-neck characteristics, where additional stent assistance is generally required to prevent coil protrusion into the parent artery. However, in cases where coil embolization was performed without stent assistance, none required rescue stent placement due to coil protrusion, owing to the TTDCs’ ability to maintain a stable tetrahedral frame. Nonetheless, the overall rate of stent-assisted embolization (71.7%) was not low. This is likely due to the small sample size and the wide-neck nature of the aneurysms in our study. Avoiding stent usage is advantageous, as it reduces the risk of peri-procedural thromboembolic complications [5]. Utilizing the TTDC to reduce the necessity for stent usage makes it plausible to anticipate a concomitant reduction in the incidence of thromboembolic complications. Therefore, a larger-scale comparative study is warranted to evaluate the rate of stent-assisted techniques between TTDCs and other coils. 

When using the TTDC for filling purposes, its characteristics were less prominent than those of conventional target detachable coils, such as the Target 360 Ultra or Target 360 Nano coils (Stryker, Fremont, CA, USA). However, the achieved packing density remained consistently favorable, irrespective of the use of a stent. This suggests that the TTDC’s aggregation tendency and softness, attributed to its braided stretch-resistant suture, likely contribute to achieving an optimal packing density. 

It is important to consider that the TTDCs showed a tendency to localize around the catheter tip during the aneurysm filling, particularly during the final stage. This behavior was more noticeable than that of the other coil types, such as the Target 360 Nano coils. Therefore, unless compartmentalization has occurred, once a stable frame covering the entire aneurysm has been established using a TTDC, it may be advantageous to utilize different coils during the final filling or finishing stages that can effectively disperse into any remaining empty spaces. 

Post-procedural MRI revealed focal dot-like lesions in 17.1% of cases, with no territorial lesions observed. This result is lower than previous studies, where post-procedural diffusion-positive lesions were found in 30–57.1% of the cases [28,29,30,31]. Diffusion-restricted lesions are attributed to the aneurysm selection step, events during the coil framing, the amount of coils used, procedural duration, the selected detachment method, and the perioperative antithrombotic agent [30]. Therefore, the lower incidence of diffusion-restricted lesions suggests a reduced number of events during the framing step, such as TTDC protrusion into the parent artery, which ultimately results in shorter procedure durations. 

Follow-up angiography revealed favorable outcomes in 95.3% of aneurysms, demonstrating the promising stability and durability of the TTDCs within the aneurysms, even when conventional coiling techniques were employed. Additionally, there were no instances of recanalization or the need for retreatment. The tetrahedral conformation appears to be consistently maintained over time without coil compaction. However, long-term angiographic studies are necessary to confirm the absence of recanalization.

This study has limitations in terms of its small sample size and the absence of long-term follow-up results. The study’s retrospective design is also a limitation. To address these limitations, large-scale prospective follow-up studies are warranted to provide comprehensive insights into the efficacy and long-term outcomes of TTDCs. Additionally, since our study only included procedures where TTDCs constituted more than 50% of the total coil length, a comparison between cases where TTDCs were used exclusively and those where TTDCs were used less frequently is not feasible, limiting the ability to evaluate the outcomes of TTDCs alone. Lastly, the absence of a control group and the lack of comparative analysis between TTDCs and other detachable coils impose additional limitations on this study. However, the primary advantage of TTDCs—a stable and firm frame—is based on the operator’s subjective experience. The outcomes of coil embolization procedures, typically used in comparative analyses, such as efficacy and safety measures, cannot transparently reflect these experiences. Nonetheless, further research is still needed to compare TTDCs versus other coils, particularly in terms of efficacy and safety.

## 5. Conclusions

In conclusion, the TTDC demonstrates promising characteristics for the treatment of small aneurysms in terms of efficacy and safety. By leveraging the advantages, either independently or in combination with other coil types, TTDCs can potentially enhance treatment outcomes. Additionally, we believe that TTDCs can potentially reduce stent usage, which is especially advantageous in terms of lowering the risk of thromboembolic complications and benefiting patients who cannot tolerate a dual antiplatelet regimen. Further clinical studies are required to validate and expand upon these findings.

## Figures and Tables

**Table 1:** Characteristics of the study participants.

Mean age (years), range	62.6 (37–76)
Total number of patients	41
Male	10 (24.4%)
Female	31 (75.6%)
Mean age of male patients, range	57.2 (51–68)
Mean age of female patients, range	63.4 (37–76)

**Table 2:** Characteristics of intracranial aneurysms.

Number of treated aneurysms	46
Ruptured aneurysm	1 (3.6%)
Location	
Internal carotid artery	25 (54.3%)
Anterior cerebral artery	9 (19.6%)
Middle cerebral artery	5 (10.9%)
Posterior circulation	7 (15.2%)
Mean maximum sac size (mm), range	4.5 (2.1–6.5)
Mean neck size (mm), range	3.3 (1.6–6.1)
Mean dome-to-neck ratio, range	1.3 (0.9–2.1)

**Table 3:** Procedural details and outcomes.

Aneurysm treatment technique	
Stent-assisted coil embolization	33 (71.7%)
Simple coil embolization	13 (28.3%)
Mean number of total coils used, range	4.7 (1–10)
Mean number of TTDCs used, range	3.9 (1–10)
Mean number of failed total coils, range	0.35 (0–2)
Mean number of failed TTDCs, range	0.19 (0–1)
Average total coil length (cm), range	20.4 (2–88)
Average tetra coil length (cm), range	17.3 (2–49)
Average tetra coil length/total coil length (%), range	88.5 (52.2–100)
Average packing density (%), range	34.7 (19.3–46.8)
Stent-assisted coil embolization (%), range	35.3 (19.3–41.5)
Simple coil embolization (%), range	33.2 (24.3–46.8)
Unplanned stent use	1 (2.2%)
Incorporated vessel	12 (26.1%)
Complete preservation	12 (100%)
Incomplete preservation	0 (0%)
Immediate angiography results	
Complete	41 (89.1%)
Neck remnant	1 (2.2%)
Aneurysm remnant	4 (8.7%)
Diffusion-restricted lesion	
No lesion	34 (82.9%)
Focal dot-like lesion	7 (17.1%)
Territorial lesion	0 (0%)
Device- or procedure-related complications	0 (0%)
Average initial follow-up interval (months), range	7.9 (5.5–12.5)
Follow-up angiography results	
Complete	40 (93.0%)
Neck remnant	1 (2.3%)
Aneurysm remnant	2 (4.7%)
Recanalization	0 (0%)
Retreatment	0 (0%)

## Data Availability

Anonymized data from this study will be made available by the corresponding author upon reasonable request.
